# Mapping of Incontinence Quality of Life (I-QOL) scores to Assessment of Quality of Life 8D (AQoL-8D) utilities in patients with idiopathic overactive bladder

**DOI:** 10.1186/s12955-014-0133-0

**Published:** 2014-08-30

**Authors:** Gang Chen, Jonathan T Tan, Kwong Ng, Angelo Iezzi, Jeffrey Richardson

**Affiliations:** Flinders Health Economics Group, School of Medicine, Flinders University, Adelaide, Australia; Allergan Pty Ltd, Level 4, 810 Pacific Highway. Gordon, NSW 217 Melbourne, Australia; Allergan Pty Ltd, Pasir Panjang, Singapore; Centre for Health Economics, Monash University, Melbourne, Australia

**Keywords:** Urinary incontinence, Overactive bladder, HRQOL, I-QOL, AQoL, Mapping, Cross-walk, Utility

## Abstract

**Background:**

The Incontinence Quality of Life (I-QOL) questionnaire is a commonly used and validated incontinence specific QOL instrument. The objective of this study is to develop an algorithm to map I-QOL to the Assessment of Quality of Life (AQoL) 8D utility instrument in patients with idiopathic overactive bladder (iOAB).

**Methods:**

I-QOL and AQoL-8D scores were collected in a survey of 177 Australian adults with urinary incontinence due to iOAB. Three statistical methods were used for estimation, namely ordinary least squares (OLS) regression, the robust MM-estimator, and the generalised linear models (GLM). Each included a range of explanatory variables. Model performance was assessed using key goodness-of-fit measures in the validation dataset.

**Results:**

The I-QOL total score and AQoL-8D utility scores were positively correlated (r = 0.50, p < 0.0001). Similarly, the three sub-scales of the I-QOL were correlated with the eight dimensions and two super-dimensions of the AQoL-8D. The GLM estimator, with I-QOL total score as the explanatory variable exhibited the best precision (MAE = 0.15 and RMSE = 0.18) with a mapping function given by AQoL-8D = exp(−1.28666 + 1.011072*I-QOL/100).

**Conclusions:**

The mapping algorithm developed in this study allows the derivation of AQoL-8D utilities from I-QOL scores. The algorithm allows the calculation of preference-based QOL scores for use in cost-utility analyses to assess the impact of interventions in urinary incontinence.

**Electronic supplementary material:**

The online version of this article (doi:10.1186/s12955-014-0133-0) contains supplementary material, which is available to authorized users.

## Introduction

Urinary incontinence (UI) is defined as any involuntary loss of urine. UI occurs at all ages, however, both prevalence and incidence of UI increase with age [1,2]. Incontinence is a significant health issue that has physical, social and economic implications on patients, as well as the broader community [3]. A study in the United States estimated the total cost associated with UI in the US as US$19.5 billion [4]; an amount that was greater than the sum of the annual direct costs of breast, ovarian, cervical and uterine cancers [5].

Idiopathic overactive bladder syndrome (iOAB) is a common cause of urinary incontinence. As defined by the International Continence Society, iOAB is characterized by the presence of urinary urgency, often with UI, increased frequency (>8 voids per day), and nocturia (ie, interruption of sleep ≥1 time/night to urinate) [6]. Urinary incontinence due to iOAB is to be distinguished from other etiologies of incontinence such as neurogenic detrusor overactivity, where bladder dysfunction is due to a known neurological condition (eg, spinal cord injury), or stress incontinence, which is the involuntary loss of urine on physical exertion.

The ability to control one’s elimination functions is a highly valued health outcome. Urinary incontinence and other iOAB symptoms have adverse impacts on multiple domains of health-related quality of life (HRQOL), affecting physical activity (eg, restrictions to daily activity due to leakage), sexual and interpersonal relationships, social interactions, and mental wellbeing [7]. The Australian Health Omnibus Survey found severe UI to be associated with a significant disutility of 0.32 based on the Assessment of Quality of Life (AQoL) and the EuroQOL five dimensions (EQ-5D) instruments [8]. Similarly, a national survey in the US, found that, after standardising for other personal characteristics, patients with UI had clinically and significantly poorer SF-36 scores across all domains compared to patients without UI [9].

The measurement and monitoring of HRQOL has become a key component for assessing the cost-effectiveness of medical interventions and health programs in clinical medicine research [10]. One common response to this need has been to include a disease specific instrument which has been specifically designed to measure dimensions of health which are important for the specific disease.

However, condition-specific measures do not provide ‘utility’ scores, which are necessary for the estimation of quality adjusted life years (QALYs) which are the unit of outcome in cost-utility analyses (CUA). CUA has become of increasing importance in the allocation of finite healthcare resources. For example, in Australia, Canada, and the United Kingdom, cost-per-QALY ratios are used by healthcare authorities in their appraisal of drug interventions. Consequently, a common approach to overcome this limitation is to develop mapping algorithms that transform condition-specific HRQOL scores into preference-based HRQOL utilities [11]. While generic utility instruments such as the SF-6D and EQ-5D can be utilised in any study regardless of the condition, this versatility comes at the expense of sensitivity to certain aspects of HRQOL that may be of importance to the conditions being studied. In the case of incontinence, a number of studies have shown that commonly used generic HRQOL instruments and multi-attribute utility instruments (MAUIs) are relatively insensitive to health dimensions that are affected by the treatment of UI [12–14]. In particular, these instruments fail to capture the full effect of poor sleep and psycho-social outcomes that are affected by incontinence. In the key study by McCallum [13], several MAUIs (ie, AQoL. EQ-5D, HUI3, SF-6D) were compared and the AQoL was identified as the instrument of choice for incontinence studies, as it provided the best coverage and sensitivity to HRQOL aspects impacted by incontinence. For this reason, the AQoL was selected for use in the present study in combination with the Incontinence Quality of Life Questionnaire (I-QOL). These two instruments are described in the Methods section.

The mapping algorithm will enable I-QOL scores observed in clinical trials for UI to be transformed into utility scores and facilitate the conduct of economic evaluations to inform decision decision-making with regards to healthcare resource allocation.

## Methods

### Data

A cross-sectional survey (N = 1341) of individuals with iOAB in Australia, Canada, France, Germany, Italy, Spain, United Kingdom and the United States was conducted between December 2012 and March 2013 [15]. In only the Australian subgroup, both the I-QOL and AQoL instruments were administered. This data has been used in the present study to develop a mapping algorithm between the I-QOL and AQoL-8D.

Patients were recruited through the Quintiles (a contract clinical research organization) patient databases and registries. These registries are a voluntary medication monitoring service that allows interested individuals to be contacted to participate in medical surveys and studies relevant to their medical conditions. Respondents were screened to ensure that only subjects ≥18 years of age, with previously diagnosed iOAB, or those who had symptoms of iOAB, were allowed to participate in the survey. Exclusion criteria included a predominance of stress UI, pregnancy, a history of neurological disorders, history of bladder disorders, including bladder stones and bladder outlet obstruction, a history of bladder or prostate cancer, and bladder reconstructive surgery.

Patient information was based on self-reported data through an online survey. Patients were granted access to the survey only after providing informed consent online. The study protocol was approved by the respective institutional review board, Ethical and Independent Review Services.

### Instruments

#### The incontinence quality of life questionnaire (I-QOL)

The I-QOL, developed by Wagner [16] and Patrick [17], is a commonly used self-reported HRQOL instrument for people with UI. The I-QOL consists of 22 items, all of which use a five-point ordinal response scale in which 1 = extremely, 2 = quite a bit, 3 = moderately, 4 = a little, and 5 = not at all.

The 22 items can be further grouped into 3 subscales: Avoidance and Limiting Behaviour (8 items), Psychosocial Impacts (9 items), and Social Embarrassment (5 items). The total I-QOL and 3 subscale scores are calculated by summing the unweighted item score and transforming them to a 100 point scale where 0 = most severe, and 100 = no problem . The instrument has been widely used and has been successfully validated for people with UI [18,19].

### Assessment of quality of life eight dimension (AQoL-8D)

Building upon the earlier AQoL MAUIs (ie, AQoL-4D and AQoL-6D), the AQoL-8D has significantly increased the content for health states pertaining to social and psychological problems [20], which are relevant in the study of incontinence. The AQoL-8D contains 35 items, these are grouped into eight dimensions (Independent Living, Relationships, Mental Health, Coping, Pain, Senses, Happiness and Self-Worth), which may be further grouped into two ‘super-dimensions’ (ie, physical and psychosocial/mental). With four to six response levels for each of the 35 items, this allows for 2.37 × 10^23^ possible health states. The time trade-off method was used for the preference-based valuation of AQoL-8D health states. Utility scores were calculated from the 35 items using the AQoL-8D scoring algorithm (Version 13), available at www.AQoL.com.au. Although AQoL-8D has not been widely used to date, it has been successfully tested for reliability and validity [21]. Recent evidence indicates that AQoL-8D has greater sensitivity to the psycho-social dimensions of QOL (which are relevant in the study of incontinence) than the other utility instruments in common use [22].

### Statistical analysis

The survey data were used to estimate a mapping algorithm between the I-QOL and AQoL-8D that can be applied for other studies. The approach is a widely used strategy in mapping analysis and is referred to in the literature as the ‘transfer to utility regression technique’ [23].In this study we adopted two model specifications. The first used I-QOL total scores and the second used the I-QOL subscale scores as the independent variable(s). Two demographic variables (age and gender) were included in the models which were specified as the following equations:

- Model 1: I-QOL total score model

$$ AQoL8D={\alpha}_0+\beta \cdot IQOL+{\alpha}_1\cdot AGE+{\alpha}_2\cdot GENDER+\mu $$

- Model 2: I-QOL subscale score model

$$ AQoL8D={\alpha}_0+{\gamma}_1\cdot IQO{L}_{PS}+{\gamma}_2\cdot IQO{L}_{SE}+{\gamma}_3\cdot IQO{L}_{ALB}+{\alpha}_1\cdot AGE+{\alpha}_2\cdot GENDER+\mu $$

where *AQoL8D* is the AQoL-8D utility; *IQOL* is the I-QOL total score; *IQOL*_*PS*_, *IQOL*_*SE*_, and *IQOL*_*ALB*_ are the scores of Psychosocial Impacts scale, Social Embarrassment scale, and Avoidance and Limiting Behaviour scale respectively; *AGE* is a continuous variable for the respondents’ age; *GENDER* is a dummy variable that equals 1 if the respondent is male and 0 otherwise; *α*_0_ is a constant, *α*_1_, *α*_2_, *β*, and *γ* are the coefficients to be estimated, *μ* is the error term. The squared terms of the I-QOL (total/scale) scores were also considered in the relevant models. In the final models, only the explanatory variables which were statistically significant (p ≤ 0.05) were retained. A stepwise regression technique with forward selection [24] was used to choose the “best” combination of predictors.

Three statistical techniques were adopted in the study to estimate the two models.

1. Firstly, an ordinary least squares (OLS) estimator was used. This has been the most widely used technique in the literature [11,23].
2. Secondly, an effective robust estimator, the MM estimator [25] was used. This has recently been proposed for mapping analysis by Chen [26]. The justification for using robust estimators lies in the fact that the OLS estimator is highly sensitive to sample outliers.
3. Lastly, the generalised linear model (GLM) was used, which allows for the non-normal distribution of dependent variables (e.g. left/negatively skewed utility scores) [27]. For the GLM estimation a choice must be made between the type of ‘family estimate’ (e.g. Gaussian, inverse Gaussian, binomial, gamma) and the link function (e.g. identity, log, logit, cloglog, log-log, log-complement, power).

Based on the goodness-of-fit results in the validation analysis, the *Gaussian* family with *log* link was chosen as the most appropriate method.

Other popular estimators that are widely adopted in the mapping analysis also include the Tobit estimator, the censored least absolute deviations (CLAD) estimator, and the Two-Part Model (2 PM) which especially take into account censoring issues (e.g. a high proportion of respondents report full health with a utility of 1) [28]. These three estimators are not used in this analysis because sample censoring is not an issue when AQoL-8D utility was scored in this study.

Model performance was assessed using the internal data, due to the lack of availability of an external validation dataset. The full sample was divided equally into five groups using computer-generated random numbers. In each group, 80% of the sample was assigned to the “estimation sample” that was used to generate the mapping algorithm, while the remaining 20% of the sample (assigned to the “validation sample”) was used to predict AQoL-8D utilities based on the above algorithm. This procedure was repeated five times, so that each of the five random groups was used in the estimation and validation exercises.

Goodness-of-fit was examined by the mean absolute error (MAE) and the root mean square error (RMSE). The best fitting model was identified as the combination with the lowest combination of MAE and RMSE values. When there was a disagreement between the optimal chosen combination, the procedure used by Kay [29] was adopted, which used the RMSE of the validation analysis as the key criteria to measure model performance. All analyses were estimated in STATA version 12.1 (StataCorp LP, College Station, Texas, USA).

## Results

### Patient characteristics

A total of 254 subjects were recruited through the Australian centres. Of these, 177 iOAB subjects with UI completed the I-QOL and AQoL instruments and were included in the present study. As shown in Table 1, the mean age of patients was 56 (SD: 14), with females comprising 85% of the samples. Participants experienced an average of 2.5 incontinence episodes per day (range 1 to 15, SD 2) and 1.9 nocturia episodes per night. The respondents had a mean I-QOL total score of 77.99 (SD 18.34, range 23.86 to 100), and a mean AQoL-8D utility of 0.62 (SD 0.21, range 0.13 to 0.99), which is comparable to utility estimates previously reported in incontinent patients in Australia [8].

**Table 1:** **General characteristics of patients**

**Patient characteristic**	**N = 177**
Mean age, years	56.5 (SD: 13.7)
Females	85.3%
Caucasian	95.7%
Mean years since diagnosis of iOAB, n (%)	
• <1 year	28 (16)
•1-4 years	105 (59)
• 5-10 years	28 (16)
• >10 years	16 (9)
History of anti-cholinergic medication use, n (%)	
• Used previously	157 (89)
• Failed	71 (40)
Education, n (%)	
• Elementary/High School	129 (73)
• College/University degree	35 (20)
• Post-graduate degree	13 (7)
Income, n (%)	
• ≤$50,000	91 (51)
• $50,001 to $100,000	35 (20)
• >$100,000	24 (14)
• Did not disclose	27 (15)
Urinary incontinence, mean episodes per day (SD)	2.5 (2.2)
Micturitions, mean episodes per day (SD)	8.5 (4.2)
Nocturia^a^, n (%)	95 (53.7)
Urgency, mean episodes per day (SD)	3.2 (3.4)
I-QOL score, mean (SD)	78.0 (18.3)
AQoL-8D utility, mean (SD)	0.62 (0.21)

**Figure 1:**
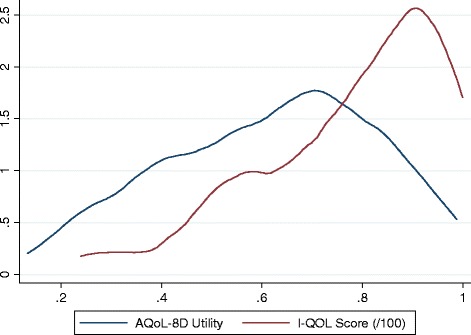
**Kernal density of AQoL-8D utility and I-QOL total score.**

The distribution of the I-QOL total score and AQoL-8D utility of the study respondents is shown in Figure 1. The correlations between I-QOL scores and AQoL dimension values and utility are presented in Table 2. Notably, all I-QOL subscales are significantly correlated with AQoL-8D dimensions (p < 0.0001). The correlation ranges from 0.22 (between Avoidance and Limiting Behaviour in I-QOL and Mental Health in AQoL-8D) to 0.47 (between Avoidance and Limiting Behaviour in I-QOL and Independent Living in AQoL-8D). Overall, the I-QOL total score and AQoL-8D utility show moderately correlation with a coefficient of 0.50.

**Table 2:** **Correlations between I-QOL and AQoL-8D**

	**I-QOL subscale scores**			**I-QOL total score**
	**Avoidance & limiting behaviour**	**Psychosocial impacts**	**Social embarrassment**	
**AQoL-8D dimensions**				
Independent Living	0.4698	0.4584	0.4186	0.4876
Relationships	0.3104	0.3377	0.2914	0.3406
Mental health	0.2222	0.2805	0.3256	0.2944
Coping	0.3082	0.3555	0.3971	0.3786
Pain	0.4289	0.3878	0.3627	0.4269
Senses	0.3371	0.3351	0.3808	0.3764
Happiness	0.3103	0.3763	0.3734	0.3806
Self-Worth	0.3597	0.4526	0.4468	0.4520
**AQoL-8D super-dimensions**				
Physical	0.4964	0.4614	0.4523	0.5089
Mental	0.3227	0.3779	0.3716	0.3854
**AQoL-8D utility**	**0.4384**	**0.4813**	**0.4668**	**0.4993**

All correlation are significant (p < 0.0001).

### Mapping results

In all of the model specifications the squared term of the I-QOL score and the age and gender variables were statistically insignificant and are not included in the results reported below. The goodness-of-fit statistics for the remaining models and statistical methods are reported in Table 3. The first three columns show the predicted mean minimum, maximum AQoL-8D utilities. With the exception of the MM-estimator, the predicted mean utilities, based on OLS estimators and GLM estimators, are either identical or very close to the observed (sample) mean utility. However, it should be noted that all predicted utilities tend to over predict the lowest, and under predict the highest boundary of the observed utility. This is not uncommon in the transformation analysis [30–32].

**Table 3:** **Goodness-of-fit results for mapping from I-QOL score to AQoL-8D utility score**

	**Mean AQoL-8D (1)**	**Min AQoL-8D (2)**	**Max AQoL-8D (3)**	**MAE (4)**	**RMSE (5)**
Observed	0.6176	0.1331	0.9888	―	―
**Method 1: OLS**					
Model 1	0.6176	0.3092	0.7430	0.1463	0.1808
Model 2	0.6176	0.2854	0.7243	0.1472	0.1806
**Method 2: MM-estimator**					
Model 1	0.6185	0.2844	0.7543	0.1465	0.1810
Model 2	0.6231	0.2644	0.7079	0.1479	0.1831
**Method 3: GLM** ^**†**^					
Model 1	0.6176	0.3516	0.7591	0.1463	0.1804
Model 2	0.6174	0.3254	0.7387	0.1461	0.1795

MAE: mean absolute error; RMSE: root mean squared error.

^†^The *Gaussian* family with *log* link was used.

The MAE and RMSE are reported in the last two columns of Table 3. The MAE ranged from 0.1461 to 0.1479, whilst the RMSE varied from 0.1795 to 0.1831. Although not reported in Table 2, the R-squared statistics for both models estimated using OLS were 0.2493 (Model 1) and 0.2509 (Model 2), which are within the acceptable range for mapping algorithms [11]. The MAE and RMSE were very similar across the various combinations of models and methods, with Model 2 (using I-QOL subscale scores as key explanatory variables) based on GLM estimator having the lowest MAE (0.1461) and lowest RMSE (0.1795).

The goodness-of-fit results from the validation analysis are reported in Table 4. As shown in columns 4 and 5, the minimum MAE was obtained using the OLS estimate with Model 1, while the best fit using the RMSE criterion was achieved with the GLM estimate on Model 1. The relative small sample size, and the small variance in MAE/RMSE between models may have led to the differing conclusions between the full sample and validation analysis. As discussed in the Methods section, when there is a disagreement, the final decision was made based on the RMSE in the validation analysis. Thus, it is recommended that the mapping algorithm, based on I-QOL total score using GLM estimator (with *Gaussian* family and *log* link), should be adopted. From Table 4 this is given in the following equation:

**Table 4:** **Goodness-of-fit results from validation analysis**

	**Mean AQoL-8D (1)**	**Min AQoL-8D (2)**	**Max AQoL-8D (3)**	**MAE (4)**	**RMSE (5)**
Observed	0.6176	0.1331	0.9888	―	―
**Method 1: OLS**					
Model 1	0.6172	0.2858	0.7574	0.1511	0.1852
Model 2	0.6186	0.2945	0.7448	0.1538	0.1882
**Method 2: MM-estimator**					
Model 1	0.6182	0.2565	0.7695	0.1516	0.1862
Model 2	0.6233	0.2742	0.7290	0.1530	0.1880
**Method 3: GLM** ^**†**^					
Model 1	0.6168	0.3291	0.7747	0.1514	0.1848
Model 2	0.6180	0.3235	0.7617	0.1536	0.1877

MAE: mean absolute error; RMSE: root mean squared error.

^†^The *Gaussian* family with *log* link was used.

$$ \mathrm{AQoL}-8\mathrm{D}\ \mathrm{utility} = \exp \left(-1.28666 + \left(1.011072*\mathrm{IQOL}/100\right)\right). $$

**Figure 2:**
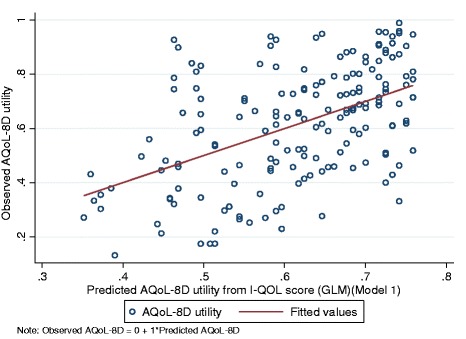
**Scatter correlation between observed and predicted AQoL-8D utilities.**

The mapping equations corresponding to the goodness-of-fit results (shown in Table 3) are reported in Table 5. The scattergram correlation between the original AQoL-8D utility and the utility predicted by the preferred model is shown in Figure 2. The STATA commands for using the algorithm is given in Additional file 1.

**Table 5:** **Mapping equations from I-QOL score to AQoL-8D utility**

	**OLS**		**MM-estimator**		**GLM** ^**‡**^	
	**Coeff.**	**SE**	**Coeff.**	**SE**	**Coeff.**	**SE**
**Model 1 (using I-QOL total score)**						
I-QOL^†^	0.5697422	0.0747355**	0.6172410	0.0730065**	1.0110720	0.1441272**
Constant	0.1732625	0.0598689**	0.1370898	0.0615673*	−1.2866600	0.1225249**
**Model 2 (using I-QOL subscale scores)**						
I-QOL-PS^†^	0.3469097	0.1252910**	0.5913308	0.0627952**	0.6703759	0.2472593**
I-QOL-SE^†^	0.1985825	0.0938378*			0.3521859	0.1691879*
Constant	0.1787669	0.0690521**	0.1165455	0.0564778*	−1.3254590	0.1526178**

SE - standard errors. *p < 0.05, **p < 0.01. ^†^The I-QOL total/subscale scores included in the regression model were calculated as original scores divided by 100. ^‡^The *Gaussian* family with *log* link was used.

I-QOL-PS: I-QOL Psychosocial Impacts; I-QOL-SE: I-QOL Social Embarrassment.

## Discussion and conclusion

With the increasing importance of health technology assessment in health system reforms and decision-making, the measurement of HRQOL are increasingly commonplace in clinical trials and public health intervention programs. However, respondent burden limits the number and type of QOL instruments included in clinical studies. Mapping analysis provides a method for transforming condition-specific quality of life scores into utilities which can be used in Cost Utility Analysis. The present study provides a mapping algorithm to transform the I-QOL total score, commonly used in incontinence clinical trials [33,34], to the AQoL-8D utility to enable the estimation of health state utility values.

In the present study, several model specifications and statistical methods were assessed. The best model result was obtained using the I-QOL total score as the key explanatory variable (Model 1), with the algorithm estimated with a robust GLM estimator (with *Gaussian* family and *log* link). As shown in Table 4, the model using the three I-QOL subscales also showed significant predictive ability. In particular, the Psychosocial Impact and Social Embarrassment subscales both showed significant associations, with the Psychosocial Impact subscale showing the strongest correlation with AQoL-8D (r = 0.48). This confirms the appropriateness of using the AQoL-8D to measure utility, as psycho-social QOL has been shown to be of importance for patients with UI and previous studies have shown AQoL-8D to be the most sensitive MAU instrument to psychosocial health among six generic preference-based instruments [22].

To date only one published study has mapped the I-QOL to preference-based HRQOL instruments (EQ-5D) [29]. The preferred model in the present study displays greater precision with both lower MAE and RMSE values (0.15 and 0.18, respectively vs. 0.17 and 0.22 in Kay *et al*.). The study by Kay [29] adopted the 2 PM statistical method, due to the ceiling effects with the use of EQ-5D where 46% of subjects reported full health. As discussed in the Methods section, 2 PM was not considered in this study as no patient in this sample had full health on the AQoL-8D scale. In turn, the larger MAE and RMSE values in the study by Kay [29] are probably attributable to the methodological disadvantage of coping with the presence of significant ceiling effects. Consistent with the results in Kay [29] age and non-linear terms of I-QOL scores were found to be insignificant (p > 0.05).

There are several limitations to this study. Firstly, the sample size is modest, although mapping studies with smaller sample sizes have been reported upon in the literature [11,23]. Nevertheless, it is desirable that further mapping studies are conducted using larger samples to test the reliability of the mapping algorithm reported here. Secondly, the model performance is validated with internal data. Since mapping is data dependent, the choice of response sample may influence the calibration of mapping algorithms. A cross-validation study should therefore be conducted when an external dataset is available. Thirdly, the predicted AQoL-8D utilities do not capture the full range of observed AQoL-8D utilities. The over-prediction of the lowest utilities and the under-prediction of the highest utilities may result in an underestimation of the utility gain.

Despite these qualifications, the goodness-of-fit measures reported in this study are within the ranges of previously published studies [11]. It is recognized that transformations cannot create information about dimensions which are not included in the instrument. As such the transformation of the I-QOL is the second best alternative to the use of an appropriate multi attribute utility instrument that is able to adequately capture changes in aspects of HRQOL affected by UI.

Results reported here indicate that the I-QOL can be mapped to AQoL-8D utility with acceptable precision at the group level in patients with urinary incontinence due to iOAB. While both aggregated and individual level predictions of AQoL-8D utilities can be incorporated within the CUA, it is recommended that the group level predicted utility is more suitable [11].

## Additional file

Additional file 1:
**STATA algorithm.**
