# Supplementary material for: Mapping of Incontinence Quality of Life (I-QOL) scores to Assessment of Quality of Life 8D (AQoL-8D) utilities in patients with idiopathic overactive bladder
Source: Health Qual Life Outcomes. 2014 Aug 30;12:133. doi: 10.1186/s12955-014-0133-0 (PMC4159518; doi:10.1186/s12955-014-0133-0)
Supplement: Additional file 1: — STATA algorithm. [file 12955_2014_133_MOESM1_ESM.doc]

**Additional file 1 STATA algorithm**

********************************************************************

** Mapping I-QOL scores onto AQoL-8D utilities

** This program generates AQoL-8D utility based on the GLM estimates on the I-QOL total score (Model 1, Table 4) through Stata Software

** Note: IQOL refers to the I-QOL total score

********************************************************************

gen IQOL_100 = IQOL/100

gen temp1 = -1.28666 + 1.011072*IQOL_100

gen IQOL_MOD1_GLM = exp(temp1) /*inverse log link*/

label var IQOL_MOD1_GLM "AQoL-8D utility, predicted by I-QOL total score (Model 1, GLM)"

drop IQOL_100 temp1

sum IQOL_MOD1_GLM

******************************The End*******************************
